# SUPPRESSION OF GHRELIN SIGNALING EXACERBATES ULCERATIVE COLITIS IN OLDER MICE

**DOI:** 10.1093/geroni/igz038.334

**Published:** 2019-11-08

**Authors:** Chia-Shan Wu, Jiyeon Noh, Ellie Tuchaai, Jennifer A DeLuca, Kimberly F Allred, Clinton D Allred, Yuxiang Sun

**Affiliations:** Texas A&M University, College Station, Texas, United States

## Abstract

The aging process is characterized by increased chronic low-grade inflammation, aka inflamm-aging, which offend is accompanied by ‘leaky gut’ syndrome. Inflamm-aging is a highly significant risk factor for both morbidity and mortality in the older adult population (>65 years of age). In addition, there is a growing prevalence of inflammatory bowel disease (IBD), a chronic inflammatory condition of the gastrointestinal tract in the older adult population. The pathogenesis of late-onset IBD is suggested to be more complex compared with younger IBD patients; the causes determining the age of IBD onset remain unexplained. Ghrelin is a 28-amino-acid peptide hormone mainly produced by X/A-like cells of the stomach, with well-characterized functions in growth hormone secretion, food intake, adiposity and insulin resistance. Ghrelin’s biological relevant receptor is Growth Hormone Secretagogue Receptor (GHS-R). Ghrelin and ghrelin mimetics have been considered viable candidates for treating cachexia, sarcopenia, and gastrointestinal disorders. As expected, we observed that the expression of tight junction proteins in colon mucosal layer decreases with age. When challenged with dextran sulfate sodium (DSS) to induce experimental ulcerative colitis, 18-months old male C57BL/6 mice exhibited exacerbated disease activity scores compared to young male mice (5-months), showing worsened pathology such as rectal bleeding and difficulty in defecation. DSS-induced colitis was exacerbated in both ghrelin-deficient (Ghrl-/-) and ghrelin receptor-deficient (Ghsr-/-) mice. Together, these data suggest endogenous ghrelin signaling contributes to susceptibility to colitis, and ghrelin signaling pathway may present a novel target for prevention and treatment of leaky gut syndrome in aging.

